# The cerebrospinal fluid biomarker ratio Aβ42/40 identifies amyloid positron emission tomography positivity better than Aβ42 alone in a heterogeneous memory clinic cohort

**DOI:** 10.1186/s13195-022-01003-w

**Published:** 2022-04-26

**Authors:** Michaela Amft, Marion Ortner, Udo Eichenlaub, Oliver Goldhardt, Janine Diehl-Schmid, Dennis M. Hedderich, Igor Yakushev, Timo Grimmer

**Affiliations:** 1 Department of Psychiatry and Psychotherapy, Klinikum rechts der Isar, Technical University of Munich, School of Medicine, Munich, Germany; 2 Clinical Development Medical Affairs, Roche Diagnostic Solutions, Roche Diagnostics GmbH, Penzberg, Germany; 3 Department of Neuroradiology, Klinikum rechts der Isar, Technical University of Munich, School of Medicine, Munich, Germany; 4 Department of Nuclear Medicine, Klinikum rechts der Isar, Technical University of Munich, School of Medicine, Munich, Germany

**Keywords:** Alzheimer’s Disease, Cerebrospinal Fluid, Biomarkers, Positron Emission Tomography, Aβ42/40 Ratio

## Abstract

**Background:**

Cerebrospinal fluid (CSF) analysis for detecting amyloid positivity may be as reliable as positron emission tomography (PET). We evaluated the performance of the amyloid beta (Aβ)42/40 ratio for predicting amyloid positivity by PET, compared with Aβ42 alone, and phosphorylated tau 181 (pTau181)/Aβ42 and total tau (tTau)/Aβ42 ratios, using fully automated CSF immunoassays (Roche Diagnostics International Ltd, Rotkreuz, Switzerland) in a heterogeneous cohort of patients with a range of cognitive disorders reflecting the typical population of a memory clinic.

**Methods:**

CSF samples from 103 patients with known amyloid PET status (PET positive = 54; PET negative = 49) were retrospectively selected from one site in Germany; 71 patients were undergoing treatment for mild cognitive impairment (*n* = 44) or mild-to-moderate dementia (*n* = 27) due to Alzheimer’s disease (AD), and 32 patients were undergoing treatment for non-AD-related cognitive disorders. Aβ42, pTau181, and tTau concentrations were measured in CSF samples using the respective Elecsys^®^ CSF immunoassays modified for use on the cobas e 411 analyzer; Aβ40 concentrations were measured using a non-commercially available robust prototype assay. Sensitivities/specificities for amyloid positivity cut-offs (Youden-derived and pre-defined) were calculated, and receiver operating characteristic analyses determined area under the curve (AUC) versus amyloid PET status. Limitations include a small sample size, use of a pre-analytical protocol not in accordance with the Elecsys CSF immunoassay method sheets, and the lack of a pre-defined cut-off for Aβ42/40.

**Results:**

Point estimates for sensitivity and specificity of CSF biomarkers and biomarker ratios versus amyloid PET were 0.93 and 0.57 for Aβ42, 0.96 and 0.69 for pTau181/Aβ42, 0.92 and 0.69 for tTau/Aβ42, and 0.94 and 0.82 for Aβ42/40. For AUCs, point estimates (95% confidence intervals) versus amyloid PET were 0.78 (0.68−0.88) for Aβ42, 0.88 (0.81−0.95) for pTau181/Aβ42, 0.87 (0.80−0.95) for tTau/Aβ42, and 0.90 (0.83−0.97) for Aβ42/40.

**Conclusions:**

CSF Aβ42/40 ratio can predict PET amyloid positivity with high accuracy in patients with a range of cognitive disorders when evaluating Aβ pathology independent of tau and neurodegeneration for research purposes. The performance of Aβ42/40 was comparable with pTau181/Aβ42 and tTau/Aβ42 used in clinical practice and better than Aβ42 alone.

## Introduction

Alzheimer’s disease (AD) is the most common form of dementia, accounting for 60−80% of all dementia syndromes [1]. The disease is defined neuropathologically by the presence of extracellular amyloid-beta (Aβ) plaques and neurofibrillary tangles of hyperphosphorylated tau protein [2, 3]. The 2018 National Institute on Aging and Alzheimer’s Association “A/T/(N)” research framework comprises several biological biomarkers for the diagnosis of AD, including low levels of cerebrospinal fluid (CSF) Aβ(1–42) (Aβ42) and cortical amyloid positron emission tomography (PET) as markers of Aβ pathology (labeled “A”); elevated CSF phosphorylated tau 181 (pTau181) and cortical tau PET as markers of fibrillar tau (labeled “T”); and CSF total tau (tTau), [^18^F]-fluorodeoxyglucose-PET hypometabolism, and atrophy on magnetic resonance imaging as markers of neurodegeneration or neuronal injury (labeled “(N)”) [4]. The presence of different markers determines each patient’s individual disease status, i.e., whether the patient has Alzheimer’s pathological change only (Aβ pathology: A^+^) or AD (Aβ and fibrillar tau pathology: A^+^T^+^) [4]. Currently, there are two methods to identify Aβ pathology in vivo. Firstly, PET measuring Aβ pathology using various Aβ binding tracers, such as [^18^F]-flutemetamol and [^18^F]-florbetapir, can be used [5]. There is high concordance between different tracers [6], which have been validated against Aβ pathology measured by histopathological samples [7, 8]. Secondly, Aβ pathology can be identified by measuring CSF Aβ42 concentration, which could provide a reliable, cost-effective, and quick alternative method to amyloid PET as a diagnostic tool [9–11].

The fully automated Elecsys^®^ β-Amyloid(1–42) CSF, Phospho-Tau (181P) CSF, and Total-Tau CSF immunoassays (Roche Diagnostics International Ltd, Rotkreuz, Switzerland) are intended for the in vitro quantitative determination of Aβ42, pTau181, and tTau, respectively, in CSF [11, 12], and are Conformité Européen approved for clinical use. The ratios of these individual biomarkers, pTau181/Aβ42 and tTau/Aβ42 (corresponding to A/T and A/(N) ratios in the A/T/(N) research framework), have demonstrated high concordance with amyloid positivity as determined by PET imaging, and both ratios have been shown to perform better at identifying Aβ pathology than using Aβ42 alone, when using the highest Youden index as the cut-off [9, 13, 14]. The pTau181/Aβ42 and tTau/Aβ42 ratios have also been shown to predict future clinical progression in patients with mild cognitive impairment (MCI) [9]. In one study, pTau181/Aβ42 and tTau/Aβ42 ratios, determined using Elecsys CSF immunoassays, demonstrated high concordance with [^18^F]-flutemetamol and [^18^F]-florbetapir amyloid PET data in two cohorts comprising patients with symptoms of cognitive impairment or AD: the Swedish BioFINDER cohort (*N* = 277; PET positive = 110/277; PET negative = 167/277; area under the curve [AUC] [95% confidence intervals (CI)]: 94.4% [91.5−97.3] for pTau181/Aβ42; 94.0% [91.0−97.0] for tTau/Aβ42, and 86.5% [82.3−90.7] for Aβ42); and the Alzheimer’s Disease Neuroimaging Initiative cohort (*N* = 646; PET positive = 347/646; PET negative = 299/646; AUC [95% CI]: 96.3% [95.2−98.0] for pTau181/Aβ42, 96.3% [94.8−97.7] for tTau/Aβ42, and 92.1% [90.0−94.3] for Aβ42). Both pTau181/Aβ42 and tTau/Aβ42 ratios outperformed Aβ42 alone [9]. The findings of the above study suggest that pTau181/Aβ42 and tTau/Aβ42 ratios determined using Elecsys CSF immunoassays have the potential to accurately identify Aβ pathology as measured by amyloid PET positivity in homogenous cohorts [9].

While using pTau181/Aβ42 and tTau/Aβ42 ratios may be considered an acceptable approach to stratify patients with AD from healthy individuals in clinical practice, it may be valuable in research settings to evaluate Aβ pathology independently when applying the A/T/(N) framework, or determining the concordance of CSF biomarkers or biomarker ratio measurements to amyloid PET positivity. To date, CSF Aβ42 alone has not achieved as high concordance rates to amyloid PET as pTau181/Aβ42 and tTau/Aβ42 ratios [9]; however, some studies have demonstrated that the Aβ42/40 ratio may be accurate in determining amyloid positivity. In 100 non-demented patients with symptoms of cognitive impairment from the Swedish BioFINDER cohort, Aβ42/40 or Aβ42/38 ratios measured using high-resolution mass spectrometry showed high concordance with [^18^F]-flutemetamol PET (AUC [95% CI]: 0.85 [0.78−0.93] for Aβ42; 0.95 [0.90−1.00] for Aβ42/40; and 0.94 [0.88−0.99] for Aβ42/38) [10]. Additionally, another study in 215 non-demented patients with symptoms of cognitive impairment showed that Aβ42/40 and Aβ42/38 ratios, measured using three immunoassays (Euroimmun, Germany], Meso Scale Discovery [Rockville, USA], and Quanterix [Billerica, USA]), predicted abnormal amyloid PET using [^18^F]-flutemetamol more accurately than Aβ42 alone; AUC values were 0.912−0.975 for Aβ42/40 and Aβ42/38 ratios, compared with 0.810−0.916 for Aβ42 alone [15]. In a retrospective study of 198 mainly cognitively normal or MCI participants, the Aβ42/40 ratio (determined using CSF assays from different manufacturers) achieved a comparable concordance with amyloid PET, compared with Aβ42 alone or pTau/Aβ42 and tTau/Aβ42 ratios calculated using the highest Youden index as the cut-off [13]. Another retrospective study of 202 predominantly cognitively normal individuals and patients with MCI or AD showed comparable concordance with amyloid PET for the CSF biomarker ratios Aβ42/40, pTau/Aβ42, and tTau/Aβ42; all ratios, calculated using the highest Youden index as the cut-off, were superior to using the individual biomarker Aβ42 alone [14]. Moreover, a retrospective study in a mixed cohort (*N* = 94) using a different automated platform showed comparable concordance with amyloid PET for the CSF biomarker ratios Aβ42/40, pTau/Aβ42, and tTau/Aβ42; all biomarker ratios, calculated using the highest Youden index as the cut-off, were superior to using the individual biomarker Aβ42 alone [16].

In this study, we evaluated the clinical performance from a research perspective of the Aβ42/40 ratio in detecting amyloid positivity as determined by PET, compared with Aβ42 alone, and the pTau181/Aβ42 and tTau/Aβ42 ratios, calculated using fully automated CSF immunoassays (Roche Diagnostics International Ltd) based on pre-defined (determined from Elecsys CSF immunoassay manufacturer instructions) and Youden-derived cut-offs. A heterogeneous cohort of patients with a range of cognitive disorders, who underwent lumbar puncture and amyloid PET, were selected for inclusion, as we aimed to understand how CSF immunoassays may perform in a broad context of pathologies reflecting the typical patient population of a memory clinic.

## Materials and methods

### Study design

This study was conducted at a single center in Munich, Germany (Centre for Cognitive Disorders, Department of Psychiatry, Klinikum rechts der Isar, Technical University of Munich, School of Medicine) between July 2019 and July 2020. Consecutive patients who underwent lumbar puncture and amyloid PET for diagnostic reasons, and for whom clinical data were available, were retrospectively enrolled in the study. Patient CSF samples, which were acquired and handled in a standardized manner, were stored frozen at −80 °C at the study center biobank prior to selection for the study.

Patients enrolled in the study were diagnosed with various cognitive disorders, including MCI due to AD [17], classified based on A/T/(N) stages (A, T, and (N) were evaluated based on amyloid PET positivity, CSF pTau181 positivity, and CSF tTau positivity, respectively) [4]; mild-to-moderate dementia due to AD [18], classified based on A/T/(N) stages; frontotemporal lobar degeneration (FTLD), classified by clinical syndrome (behavioral variant frontotemporal dementia [bvFTD] [19], semantic variant of primary progressive aphasia [svPPA], or non-fluent variant of primary progressive aphasia [nfvPPA] [20]); MCI of unclear (non-AD) origin; depression; alcohol dependence; and subjective cognitive decline (SCD).

### Ethical statement

The study was submitted to and approved by the Ethics Committee of the Technical University of Munich, Munich, Germany (Project Code: 312/19S). All participants provided written consent for the research use of their data and the study was performed according to the principles of the Declaration of Helsinki.

### CSF collection and analysis

CSF samples were acquired by lumbar puncture, between the L3/L4 or L4/L5 intervertebral space, using atraumatic cannulas and collected in sterile polypropylene tubes. Immediately after collection, CSF samples were centrifuged at 2000 x g for 10 minutes at 4 °C to discard cells. Aliquots of CSF supernatant were frozen and stored at −80 °C at the study center biobank, prior to measurement with the CSF immunoassays. The site-specific pre-analytical protocol did not fully adhere to the CSF immunoassay method sheets as the CSF collection procedure, type of polypropylene tubes (Sarstedt 13 mL) used, and size of the aliquots (0.5 mL) differed.

Concentrations of Aβ42, pTau181, and tTau were measured in patient CSF samples using the respective Elecsys CSF immunoassays, which had been modified for use for research purposes on the cobas e 411 analyzer. CSF concentrations of Aβ40 were measured using a robust prototype assay that is available for investigational use only. These assays (all Roche Diagnostics International Ltd, Rotkreuz, Switzerland) are fully automated electrochemiluminescence immunoassays, which utilize monoclonal antibodies in the form of a sandwich test principle. CSF samples were tested for amyloid positivity by calculating pTau181/Aβ42, tTau/Aβ42, and Aβ42/40 biomarker ratios from the corresponding measurements recorded for Aβ42, Aβ40, pTau181, and tTau concentrations.

### PET data

PET scans were conducted at the Department of Nuclear Medicine, Technical University of Munich (Munich, Germany) on a Siemens Biograph mMR or Biograph mCT (Erlangen, Germany), using one of three different PET tracers: [^11^C]-Pittsburgh Compound B, [^18^F]-florbetaben, and [^18^F]-florbetapir. Amyloid PET positivity was visually assessed following United States Food and Drug Administration-approved algorithms by two independent raters who were blinded to each other and to the clinical information. In the event of discordant readings (*n* = 2), a case discussion was initiated between the two raters, and consensus was achieved.

### Statistical analyses

All samples were verified by the four-eyes principle, whereby every entry is checked against the source data by two individuals. All data were analyzed using the statistical platform software IBM SPSS Statistics Version 26. No missing data were reported.

Amyloid PET positivity was used as the reference method. The concordance of CSF biomarker analysis with amyloid PET positivity was calculated based on Youden-derived cut-offs and pre-defined cut-offs, which were determined from Elecsys CSF immunoassay manufacturer instructions. The pre-defined cut-offs for Aβ42 (< 1000 pg/mL), pTau181/Aβ42 ratio (> 0.024), and tTau/Aβ42 ratio (> 0.28) were previously established in patients with MCI or SCD with suspected AD and developed using a modified site-specific pre-analytical protocol [9]. For Aβ42/40 ratio, a pre-defined cut-off has not been established; therefore, a Youden-derived cut-off of 0.048 was used. Receiver operating characteristic (ROC) analyses were conducted; AUCs were calculated and compared using the ROC Analysis feature in SPSS (SPSS Statistics Base).

Scatterplots of Aβ42 versus Aβ40, pTau181, and tTau were used to further evaluate the biomarker ratios and their ability to differentiate between amyloid PET-positive and -negative individuals.

## Results

### Patient characteristics

A total of 103 patients were enrolled in the study (male, *n* = 46 [44.66%]; mean age [standard deviation], 66.37 [± 9.75] years): 44 (42.72%) patients were diagnosed with MCI due to AD; 27 (26.21%) patients with mild-to-moderate dementia due to AD; 17 (16.50%) patients with FTLD (bvFTD: *n* = 6; svPPA: *n* = 3; nfvPPA: *n* = 8); eight (7.77%) patients with MCI of unclear origin; five (4.85%) patients with depression; one (0.97%) patient with alcohol dependence; and one (0.97%) patient with SCD (Table 1). Fifty-four (52.43%) patients showed visual amyloid PET positivity, and 49 (47.57%) amyloid PET negativity.

**Table 1:** Patient characteristics

Patients, ***N***	103
Male, *n* (%)	46 (44.66)
Female, *n* (%)	57 (55.34)
Mean age ± SD, years (range)	66.37 ± 9.75 (43–84)
Median interval between PET and CSF, days (IQR)	41 (16–94)
PET positive, *n* (%)	54 (52.43)
PET negative, *n* (%)	49 (47.57)
PET tracer, *n* (%)	
^11^C-PiB	67 (65.05)
F18-Florbetaben	14 (13.59)
F18-Florbetapir	22 (21.36)
Diagnosis	
MCI due to AD, *n* (%)	**44 (42.72)**
PET positive^a^	27 (61.36)
High likelihood^a, b^	15 (34.09)
Unlikely due to AD^a^	12 (27.27)
Conflicting/uninformative^a^	17 (38.63)
*AT(N) pathology*^a^	
A+T-(N)-	10 (22.72)
A+T+(N)-	2 (4.55)
A+T+(N)+	15 (34.09)
A+T-(N)+	0 (0)
A-T+(N)+	5 (11.36)
A-T+(N)-	0 (0)
A-T-(N)+	0 (0)
A-T-(N)-	12 (27.27)
Mild/moderate dementia due to AD, *n* (%)	**27 (26.21)**
PET positive^a^	23 (85.19)
High biomarker probability^a^	18 (66.66)
Uninformative^a^	9 (33.33)
*A/T/(N) pathology*^a^	
A+T-(N)-	5 (18.52)
A+T+(N)-	1 (3.70)
A+T+(N)+	17 (62.96)
A+T-(N)+	0 (0)
A-T+(N)+	3 (11.11)
A-T+(N)-	0 (0)
A-T-(N)+	0 (0)
A-T-(N)-	1 (3.70)
FTLD, *n* (%)	**17 (16.50)**
PET positive^a^	2 (11.77)
bvFTD^a^	6 (35.29)
svPPA^a^	3 (17.65)
nfvPPA^a^	8 (47.06)
Other, *n*^c^ (%)	**15 (14.56)**
PET positive^a^	2 (13.33)

*A* Aβ pathology based on amyloid PET positivity, *AD* Alzheimer’s disease, *bvFTD* behavioral variant frontotemporal dementia, *CSF* cerebrospinal fluid, *FTLD* frontotemporal lobar degeneration, *IQR* interquartile range, *MCI* mild cognitive impairment, *(N)* neuronal injury based on CSF tTau positivity, *nfvPPA* non-fluent variant of primary progressive aphasia, *PiB* Pittsburgh Compound B, *PET* positron emission tomography, *SD* standard deviation, *svPPA* semantic variant of primary progressive aphasia, *T* tau pathology based on CSF pTau181 positivity

^a^ Percentages calculated based on patient subgroup as the denominator; ^b^ Criteria for likelihood based on recommendations from the National Institute on Aging-Alzheimer’s Association workgroups on diagnostic guidelines for Alzheimer’s disease [18]; ^c^ There were eight patients diagnosed with MCI of unclear origin, five with depression, one with alcohol dependence, and one with subjective cognitive decline

### Concordance of CSF analysis with amyloid PET

Concordance of the individual CSF biomarkers and biomarker ratios with amyloid PET status (reference method PET positive: *n* = 54; PET negative: *n* = 49) are shown in Table 2. Using the pre-defined cut-offs, Aβ42 concordance with amyloid PET positivity achieved high sensitivity (0.93), but low specificity (0.57). As expected, sensitivity and specificity point estimates for pTau181/Aβ42 ratio (0.96 and 0.69, respectively) were higher than that for Aβ42 alone; whereas sensitivity for tTau/Aβ42 ratio (0.92) was comparable with Aβ42 alone, but specificity was considerably higher (0.69). For the Aβ42/40 ratio, the Youden-derived cut-off resulted in sensitivity and specificity point estimates of 0.94 and 0.82, respectively.

**Table 2:** Concordance of Elecsys CSF immunoassay biomarkers and biomarker ratios with amyloid PET

CSF biomarker	AUC (95% CI)	Cut-off		Sensitivity	Specificity
Aβ42	0.78 (0.68−0.88)	Pre-defined	< 1000 pg/mL	0.93	0.57
		Youden index	< 1070 pg/mL	1.00	0.57
pTau181	0.82 (0.73−0.90)	Pre-defined	> 27 pg/mL	0.69	0.80
		Youden index	> 22 pg/mL	0.83	0.71
tTau	0.78 (0.68−0.87)	Pre-defined	> 300 pg/mL	0.63	0.73
		Youden index	> 241 pg/mL	0.87	0.63
pTau181/Aβ42 ratio	0.88 (0.81−0.95)	Pre-defined	> 0.024	0.96	0.69
		Youden index	> 0.027	0.96	0.76
tTau/Aβ42 ratio	0.87 (0.80−0.95)	Pre-defined	> 0.28	0.92	0.69
		Youden index	> 0.33	0.91	0.78
Aβ42/40 ratio	0.90 (0.83−0.97)	Pre-defined	NA	NA	NA
		Youden index	< 0.048	0.94	0.82
pTau181/(Aβ42/40) ratio	0.87 (0.80−0.94)	Pre-defined	NA	NA	NA
		Youden index	> 447	0.92	0.76
tTau/(Aβ42/40) ratio	0.86 (0.79−0.94)	Pre-defined	NA	NA	NA
		Youden index	> 5164	0.94	0.73

*Aβ* amyloid beta, *AUC* area under the curve, *CI* confidence interval, *CSF* cerebrospinal fluid, *NA* not applicable, *PET* positron emission tomography, *pTau181* phosphorylated tau 181, *tTau* total tau

**Fig. 1:**
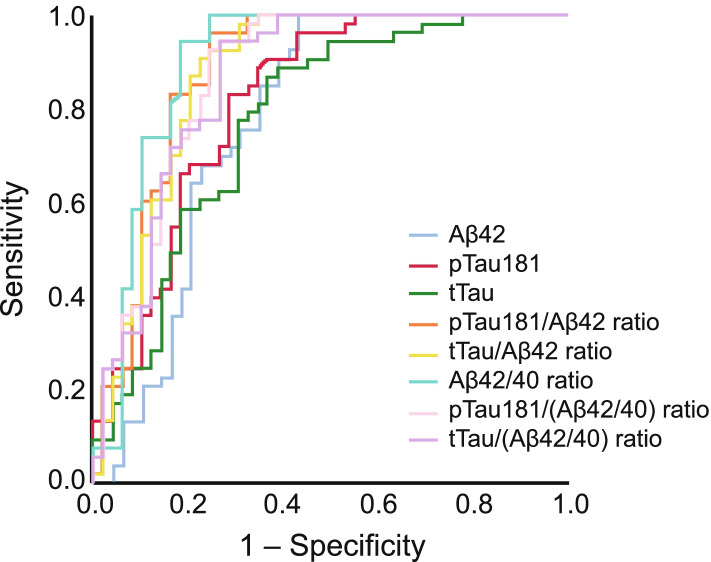
ROC analysis for CSF biomarkers and biomarker ratios versus amyloid PET positivity. Aβ, amyloid beta; CSF, cerebrospinal fluid; PET, positron emission tomography; pTau181, phosphorylated tau 181; ROC, receiver operating characteristic; tTau, total tau

Aβ42/40, pTau181/Aβ42, and tTau/Aβ42 ratios demonstrated significantly higher AUC values (0.90 [95% CI: 0.83–0.97], 0.88 [95% CI: 0.81–0.95], and 0.87 [95% CI: 0.80–0.95], respectively; pairwise comparisons: *P* = 0.004, *P* = 0.011 and *P* = 0.007, respectively) than Aβ42 alone (0.78 [95% CI: 0.68–0.88]), with Aβ42/40 ratio exhibiting the highest AUC point estimate (Table 2; Fig. 1). Therefore, the Aβ42/40 ratio showed higher concordance with amyloid PET than Aβ42 alone, and showed comparable concordance with pTau181/Aβ42 and tTau/Aβ42 ratios; adding pTau181 or tTau to Aβ42/40 did not improve concordance with amyloid PET (AUC [95% CI]: 0.87 [0.80–0.94] and 0.86 [0.79–0.94], respectively).

**Fig. 2:**
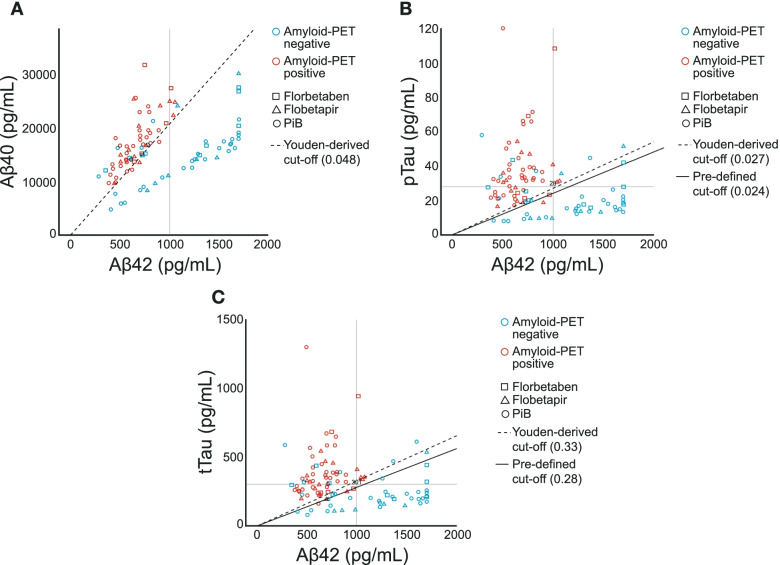
CSF Aβ42 versus (A) Aβ40, (B) pTau181, and (C) tTau for differentiating amyloid PET status*. *Amyloid PET-positive patients (*n* = 54), amyloid PET-negative patients (*n* = 49). [^11^C]-PiB, [^11^C]-Pittsburgh Compound B; Aβ, amyloid beta; CSF, cerebrospinal fluid; PET, positron emission tomography; pTau181, phosphorylated tau 181; tTau, total tau

All CSF biomarker ratios (Aβ42/40, pTau181/Aβ42, and tTau/Aβ42) demonstrated robust differentiation between amyloid PET-positive and -negative patients (Fig. 2).

## Discussion

Both imaging and CSF biomarkers have been identified as valid diagnostic tools in the most recent A/T/(N) research framework as the focus of diagnostic techniques for AD shifts from confirming the presence of symptomatic AD to identifying patients at an earlier stage of the disease [4]. Previous studies evaluating the performance of Elecsys CSF immunoassays approved for clinical use have shown high concordance for pTau181/Aβ42 and tTau/Aβ42 ratios with amyloid PET in different cohorts and demonstrated that these ratios perform better than the individual biomarkers alone [9, 13, 14].

In this study, we evaluated the performance of fully automated, research-use CSF immunoassays to determine whether they perform as well as the Elecsys CSF assay versions approved for clinical use. All individual CSF biomarkers and biomarker ratios demonstrated good concordance with amyloid PET for the detection of amyloid positivity in a heterogeneous cohort of patients with a broad range of cognitive disorders, extending on previous evidence in more homogeneous cohorts. Our results showed that the CSF Aβ42/40 ratio had higher concordance with amyloid PET than Aβ42 alone and demonstrated comparable performance to the pTau181/Aβ42 and tTau/Aβ42 ratios. The Aβ42/40 ratio also demonstrated robust differentiation between amyloid PET-positive and PET-negative patients, which was comparable to that observed using pTau181/Aβ42 and tTau/Aβ42 ratios.

Consistent with Janelidze et al., our findings suggest that the CSF Aβ42/40 ratio may better differentiate amyloid-positive from amyloid-negative individuals compared with Aβ42 alone [15]. The high concordance of the Aβ42/40 ratio to amyloid PET observed in our study is comparable to previous studies that used mass spectrometry, platforms other than those used here, or cohorts including healthy controls and patients with AD [10, 13–15].

A possible reason for the CSF Aβ42/40 ratio outperforming Aβ42 in our study, which was attributable primarily to a higher specificity, is the normalization of interpatient variability in Aβ production, as the level of Aβ peptide production differs between individuals. As a result, it is possible to obtain false-positive results if only Aβ42 is assessed. The Aβ42/40 ratio may compensate for this by assessing two Aβ peptides, of which the latter, Aβ40, is less affected by AD [10, 13–15, 21]. Additionally, the Aβ42/40 ratio is considered to be more robust than Aβ42 alone against various pre-analytical influences that can lead to false-positive results [22].

By using ratios of one protein to any other brain-derived protein, physiological fluctuations might be compensated for [9]. A/(N) ratios may aid in the normalization of patient differences in Aβ production and increase the robustness of CSF biomarkers [23]; however, the level of tTau in CSF represents the severity of neurodegeneration only, which is not specific to AD [23]. tTau can also be elevated in other neurological diseases, such as Creutzfeldt-Jakob disease or following a stroke [24, 25]. Therefore, determining the tTau/Aβ42 ratio may result in an inaccurate diagnosis of AD; for example, determining tTau/Aβ42 ratio in patients with MCI may result in a false-positive diagnosis of AD as a result of elevated tTau, despite normal levels of Aβ. CSF Tau levels are dependent on the stage of the disease and the rate of disease progression, which may lead to errors in CSF biomarker interpretation as these associations are inconsistent and not yet fully understood [26, 27]. Moreover, studies have demonstrated that almost half of AD patients do not exhibit abnormal tau, nor elevated CSF tTau [27].

In agreement with previous studies [9, 10], our findings suggest that CSF biomarker analysis may be a valid alternative to PET for the prediction of amyloid positivity in the diagnosis of a range of cognitive disorders, including AD. In general, using CSF analysis for the diagnosis of AD would reduce the time and expense spent on PET examinations. Patients would not be exposed to PET radioactivity and CSF assessments could take place at non-specialized centers [9], which would increase patient accessibility to accurate diagnosis of AD. Moreover, CSF analysis could allow patients to be easily assessed and categorized in accordance with the A/T/(N) research framework (e.g., using fully automated CSF immunoassays to enable assessment of A/T/(N) from the same patient sample) [4], which would aid the diagnosis of patients with AD, particularly those who may be asymptomatic, and the identification of patients suitable for disease-modifying treatments, such as anti-amyloid immunotherapies. On the other hand, amyloid PET examinations not only determine the presence of amyloid positivity in patients, but also quantify the regional amyloid load in the brain, which may be desirable to evaluate the effect of novel anti-amyloid treatments that are currently in development for AD [28]. Therefore, CSF biomarkers may offer an alternative tool to PET in predicting amyloid positivity, but only for global amyloid quantification in the diagnostic pathway of a range of cognitive disorders, including AD.

### Limitations

One strength of our study is the heterogeneous patient population. Patients with a variety of diagnoses were enrolled, reflecting the situation encountered in the routine setting of a memory clinic. Limitations include the relatively small sample size and the use of samples from the study center biobank, which were acquired and handled in a standardized manner, but not in accordance with the pre-analytical protocol recommended in Elecsys CSF immunoassay method sheets. This posed the risk of affecting the pre-defined assay cut-off values, as well as assay performance; for example, we observed coefficients of variation that were higher than expected. Additionally, a pre-defined cut-off has not been established for the Aβ42/40 ratio; therefore, we used a Youden-derived cut-off that was specific to the cohort of patients enrolled. This posed the risk of introducing an element of bias to the clinical performance of the assays.

## Conclusion

We demonstrate that the CSF Aβ42/Aβ40 ratio measured using fully automated, research-use CSF immunoassays can predict amyloid positivity as determined by PET with high accuracy in patients with a broad range of cognitive disorders reflecting a typical heterogeneous population of a memory clinic. The performance of the Aβ42/40 ratio in detecting amyloid positivity by PET was higher than using Aβ42 alone and comparable with the performance of the pTau181/Aβ42 and tTau/Aβ42 ratios. Our results add to previously reported literature demonstrating the strong clinical performance of pTau181/Aβ42 and tTau/Aβ42 ratios in detecting amyloid positivity in homogenous cohorts. Taken together, these findings suggest CSF biomarker analysis could provide a cost-effective alternative to PET in the AD diagnostic pathway.

## Data Availability

The datasets generated and/or analyzed during this study are available from the corresponding author on reasonable request. However, due to the nature of pseudonymized patient data, a material transfer agreement is required to meet ethical standards and data privacy laws of Germany.
